# Supplementary material for: Saving energy in turbulent flows with unsteady pumping
Source: Sci Rep. 2023 Jan 23;13:1299. doi: 10.1038/s41598-023-28519-x (PMC9871000; doi:10.1038/s41598-023-28519-x)
Supplement: Supplementary file 1 — Supplementary Information 1. [file 41598_2023_28519_MOESM1_ESM.pdf]

Supplementary material for the paper

Saving Energy in Turbulent Flows with Unsteady Pumping

Giulio Foggi Rota[1,2], Alessandro Monti[1], Marco E. Rosti[1*], Maurizio Quadrio[2*]

[1] Complex Fluids and Flows unit, Okinawa Institute of Science and Technology Graduate University, 1919-1 Tancha, Onna-son, Okinawa 904-0495, Japan
[2] Dipartimento di Scienze e Tecnologie Aerospaziali, Politecnico di Milano, via La Masa 34, 20156 Milano, Italy
[*]Corresponding authors: marco.rosti@oist.jp, maurizio.quadrio@polimi.it

To assess the robustness of the results, multiple checks have been carried out throughout our demanding numerical study. Higher grid resolutions, up to a maximum of $\Delta x^+ = 2.2,\ \ \Delta z^+ = 1.1,\ \ \Delta y^+ = 0.09 - 1.2$, have been tested to verify that the flow physics observed is independent from the discretisation adopted. The simulation yielding the most promising outcome has also been repeated on a coarser mesh ($\Delta x^+ = 13.2,\ \ \Delta z^+ = 6.6,\ \ \Delta y^+ = 0.6 - 4.1$, roughly half of the grid points in each direction). The number of forcing periods adopted in the main study was not varied, to keep the time-averaging error at a constant level. The variation in the computed energy savings has been found to be lower than 1%, thus verifying the convergence and the grid-independence of the results. Furthermore, the computational box has been proven large enough to contain the largest flow structures.
For a quantitative assessment of the performance, the proper figures of merit should be carefully designed, as a clearly defined reference flow to compare with is lacking. In this work, we follow the approach proposed by Frohnapfel, Hasegawa & Quadrio (Money versus time: Evaluation of flow control in terms of energy consumption and convenience. Journal of Fluid Mechanics, 700, 406-418 (2012)) to set up a rational comparison. The averaged energy E per unit area spent to transfer a unit mass of fluid through an infinitesimal section of the channel is

$$E = \lim_{n\to\infty} \frac{-2h\int_{nT}\Pi(t)U_b(t)dt}{2h\rho\int_{nT}U_b(t)dt}$$

where n indicates the n-th pumping cycle, h is the channel half-width, T the pulsation period, $\Pi(t)$ the instantaneous streamwise pressure gradient, $U_b(t)$ the instantaneous bulk velocity and ρ the fluid density. The averaged bulk velocity, similarly, is

$$U_b = \lim_{n\to\infty} \frac{1}{nT}\int_{nT} U_b(t)dt.$$

By adopting a turbulent friction law (e.g. the Blasius correlation), the energy E_u needed to attain the same bulk velocity $U_{b,c}$ of the controlled flow with an uncontrolled turbulent flow under

steady forcing can be computed. The subscripts u and c denote, respectively, the uncontrolled and controlled flow cases. Alternatively, the bulk velocity $U_{b,u}$ of an uncontrolled turbulent flow under steady forcing leading to the energetic consumption E_c of the controlled flow can be computed. Interpreting h/U_b as a measure of time, we can therefore define the energy and time savings, respectively, as $S = (E_u - E_c)/(E_u)$ and $(h/U_{b,u} - h/U_{b,c})/(h/U_{b,u})$: these are the quantities reported in the main text of this paper, and fully account for the energy spent to accelerate the flow during the pulsed operation of the pump.
When computing E and U_b with a DNS-based numerical experiment, the number of periods available to compute temporal averages is finite. They key random variable affecting convergence of the estimate of the mean saving is the duration of the quasi-laminar phase after pumping is stopped. In the nth pumping period, the quasi-laminar flow phase lasts for a time τ_n, measured from the end of the acceleration to the appearance of the kink in the time history of the flow rate. The collection of τ_n measured in one simulation is a single realisation of the discrete-time stochastic process τ. Over a total of 35 periods, we compute a mean value $\overline{\tau}/T = 0.18$ and a coefficient of variation $CV_\tau = 0.23$. τ_n correlates well with the cycle-averaged energy saving S_n achieved by our control over each forcing period, thus corroborating our physical understanding of the flow dynamics and of the origin of the savings. In figure 1 we plot the time histories of τ_n (panel a) and of S_n (panel b) for all the periods of the simulation run at the highest resolution. Panel c also includes data from a lower-resolution simulation, to demonstrate grid independence. Given the large variance of τ, a robust measurement of the overall energy savings S requires simulations extended over a large number of periods, with the computational burden growing accordingly. After 35 periods, results have reached statistical convergence. As visible in panel c of figure 1, the running value of S computed at the end of each period is nearly stablized, with a relative fluctuation among the last two periods lower than 3%.

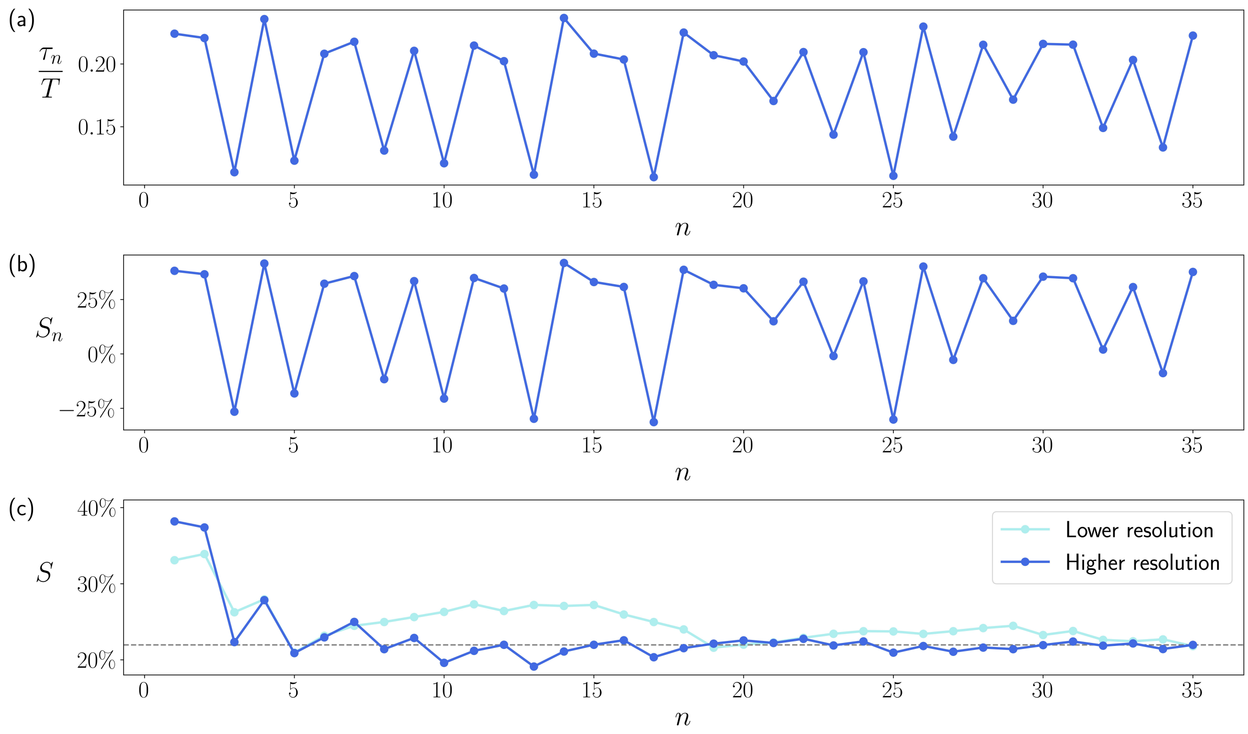

Figure 1: **History and convergence of the energy savings**: we plot the duration of the quasi-laminar flow phase $\frac{\tau_n}{T}$ (a) and the energy savings S_n over each forcing period (b), accompanied by the the value of a running average of the energy savings S achieved at the end of each period from the beginning of the simulation (c). The blue lines refer to the highest resolution considered in the study, the turquoise line to the lower one. τ_n correlates well with S_n, confirming that a longer quasi-laminar flow phase yields higher energy savings. Furthermore, the minimal fluctuations of S over the last periods of the forcing (less than 3% in relative terms) ensure that its value has reached statistical convergence.
