# Supplementary material for: Saving energy in turbulent flows with unsteady pumping
Source: Sci Rep. 2023 Jan 23;13:1299. doi: 10.1038/s41598-023-28519-x (PMC9871000; doi:10.1038/s41598-023-28519-x)
Supplement: Supplementary file 2 — Supplementary Information 2. [file 41598_2023_28519_MOESM2_ESM.pdf]

Legends of the supplementary videos for the paper

Saving Energy in Turbulent Flows with Unsteady Pumping

Video 1 (video1_*transitionSlice.m4v)*:

Transition: video of the transition from the quasi-laminar to the fully-turbulent flow phases shown in Figure 2 of the Manuscript.

Video 2 (video2_*decaySlice.m4v)*:

Decay: movie of the turbulent decay shown in Figure 2 of the Manuscript.
